# SAND: a comprehensive annotation of class D β-lactamases using structural alignment-based numbering

**DOI:** 10.1128/aac.00150-25

**Published:** 2025-05-27

**Authors:** Fedaa Attana, Soobin Kim, James Spencer, Bogdan I. Iorga, Jean-Denis Docquier, Gian Maria Rossolini, Mariagrazia Perilli, Gianfranco Amicosante, Alejandro J. Vila, Sergei B. Vakulenko, Shahriar Mobashery, Patricia Bradford, Karen Bush, Sally R. Partridge, Andrea M. Hujer, Kristine M. Hujer, Robert A. Bonomo, Shozeb Haider

**Affiliations:** 1 UCL School of Pharmacy, University College London 371646, London, United Kingdom; 2 School of Cellular and Molecular Medicine, University of Bristol 152329 , Bristol, United Kingdom; 3 Institut de Chimie des Substances Naturelles, Université Paris-Saclay 27048, Gif-sur-Yvette, France; 4 Dipartimento di Biotecnologie Mediche, Università di Siena 574285 , Siena, Italy; 5 Department of Experimental and Clinical Medicine, University of Florence 415681, Florence, Italy; 6 Department of Biotechnological and Applied Clinical Sciences, University of L’Aquila, L’Aquila, Italy; 7 Instituto de Biología Molecular y Celular de Rosario (CONICET IBR -UNR) 63031 , Rosario, Argentina; 8 Department of Chemistry and Biochemistry, University of Notre Dame 6111, Notre Dame, Indiana, USA; 9 Antimicrobial Development Specialists, LLC, Nyack, New York, USA; 10 Department of Biology, Indiana University 1772, , Bloomington, Indiana, USA; 11 Sydney School of Medicine, The University of Sydney 7799, , Sydney, New South Wales, Australia; 12 Department of Medicine, Case Western Reserve University School of Medicine 12304, , Cleveland, Ohio, USA; 13 Louis Stokes Cleveland Department of Veterans Affairs Medical Center 20083, Cleveland, Ohio, USA; 14 Department of Molecular Biology and Microbiology, Case Western Reserve University School of Medicine 12304, , Cleveland, Ohio, USA; 15 Departments of Pharmacology, Biochemistry, and Proteomics and Bioinformatics, Case Western Reserve University School of Medicine 12304, Cleveland, Ohio, USA; 16 CWRU-Cleveland VAMC Center for Antimicrobial Resistance and Epidemiology (Case VA CARES), Cleveland, Ohio, USA; 17 University of Tabuk (PFSCBR) 125900, Tabuk, Saudi Arabia; 18 UCL Centre for Advanced Research in Computing, University College London 4919, , London, United Kingdom; University of Fribourg, Fribourg, Switzerland

**Keywords:** class D, OXA, β-lactamases, SAND, structure-based sequence alignment, secondary structure annotation

## Abstract

Class D β-lactamases are a diverse group of enzymes that contribute to antibiotic resistance by inactivating β-lactam antibiotics. Examination of class D β-lactamases has evolved significantly over the years, with advancements in molecular biology and structural analysis providing deeper insights into their mechanisms of action and variation in specificity. However, one of the challenges in the field is the inconsistent residue numbering and secondary structure annotation across different studies, which complicates the comparison and interpretation of data. To address this, we propose SAND—a standardized naming system for both residues and secondary structure elements, based on a comprehensive structural alignment of all documented sequences and experimentally obtained crystal structures of class D β-lactamases. This unified framework will streamline cross-study comparisons and enhance data interpretation. Moreover, the standardized framework will enable AI-driven natural language processing (NLP) techniques to efficiently mine and compile relevant data from scientific literature, speeding up the discovery process and contributing to more rapid advancements in β-lactamase research.

## INTRODUCTION

### Consensus residues numbering

Class D β-lactamases (DBLs) form a large family with nearly 1,300 identified members in the β-Lactamase Database (Fig. S1, www.bldb.euhttp://www.bldb.eu/) (1). They are produced mainly by Gram-negative bacteria, with some by Gram-positive bacteria (2). DBLs are an important mechanism of resistance to β-lactam antibiotics, including carbapenems, one of last resort treatments to infections in hospitalized patients (3). The production of class D β-lactamases is a major public health concern, as they can often transfer between different bacterial species, allowing resistance to spread quickly and widely. Some members were found to be membrane-bound and are secreted via outer membrane vesicles (OMVs) to contribute further in antimicrobial resistance dissemination (4). Their mechanism of action involves a catalytic serine, as in classes A and C β-lactamases, since all share the conserved SXXK tetrad and KXG triad (3).

**Fig 1:**
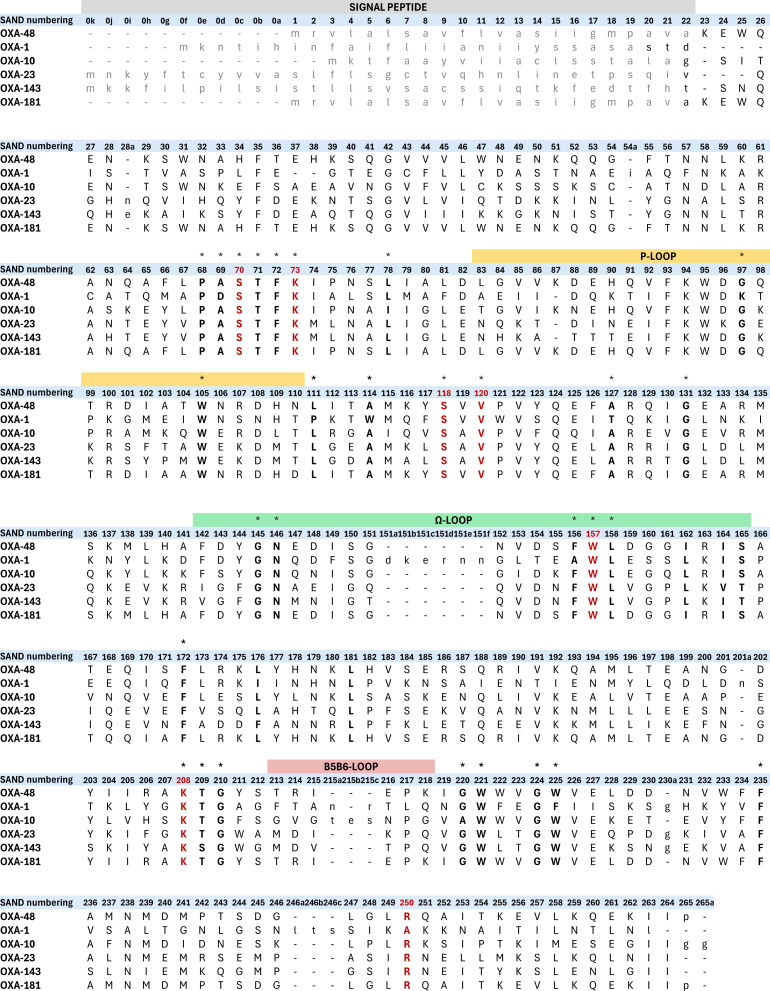
Example of SAND assigned to a query sequence. Sequence alignment of OXA-1, OXA-10, OXA-23, OXA-143, and OXA-181 to OXA-48 (the reference sequence). Signal peptide, which is removed post-translationally during export to the bacterial periplasm, is assigned using Deepsig (5) and colored in gray. Loops framing the active site are mapped above the corresponding residue numbers. Residues involved in catalytic activity are highlighted in red. Positions identified to be highly conserved, based on alignment of the latest set of DBLs listed on bldb.eu, are highlighted with an asterisk on top.

Class D enzymes have attracted molecular research to resolve their 3D structures and understand their mechanism of action that uniquely involves carbamylation of the active site lysine (6, 7). Several mutations were introduced to identify the residues essential for function and enzyme stability (8–10). Recently, a new mechanism of β-lactam deactivation via β-lactone formation was described by Aertker and Lohan in their studies on OXA-48 and OXA-23 as well as their V120L and V128L mutants, respectively (11, 12). Moreover, publications related to the design of novel β-lactamase inhibitors continue to grow in number (13–16). Notably, all the studies cited above have used the “sequential” residue numbering for each of the individual enzymes studied, although there was a proposal of a consensus numbering for class D β-lactamases (DBL numbering) by Couture et al. in 1991 (17). At the time, only five class D enzymes were known, and numbering was based on multiple sequence alignment, with Couture et al. assigning numbers to the positions in the consensus alignment without counting gaps. However, the number of known class D enzymes now exceeds 1,300, with sequence similarity of as little as 14.4% (1). Therefore, we propose an updated numbering system that accommodates longer insertions and is easy to implement by early researchers in order to guarantee assigning the same numbering to homologous residues in upcoming publications.

## MATERIALS AND METHODS

In this work, we performed structure-based sequence alignment with the UCSF ChimeraX (18) to superimpose and align representative structures from each class D subfamily listed in the Protein Data Bank (a total of 21 enzymes) (19). The MatchMaker module implemented in ChimeraX employs the commonly used Dictionary of Secondary Structure of Proteins (DSSP) algorithm to assign residues to α-helices and β-strands based on hydrogen-bonding patterns (20). The resulting alignment was manually optimised and then used to construct a Hidden Markov Model (HMM) profile via the HMMER suite, a widely used tool for creating profiles based on sequence alignments (21). A list of structures is provided in Table ST2 and residue-by-residue alignment in Table ST3. We selected OXA-48, an extensively studied carbapenemase, as the reference sequence for assigning standardized residue numbers. This sequence was chosen because it naturally features the catalytic serine residue at position 70, aligning with previous literature that adopted the same consensus of class A β-lactamases. (22)

The HMM profile enables alignment of query sequences with the OXA-48 reference. Homologous residues in the query sequence are aligned to the consensus, and each residue is assigned its standard number. Insertions relative to OXA-48 are labeled with appended lowercase letters (e.g., 216a, 216b), while deletions will result in numbers being skipped (See example in Fig. 1). This convention has been applied to and is consistent with β-lactamases of classes A, B, and C (23–25). To facilitate the adoption of the scheme by researchers from all backgrounds, we automated the process to run via a Python-based workflow that can be accessed online using Google Colab (26). A detailed tutorial guide has been included in the Supplementary Information S4). Alternatively, the workflow can be run locally using a Jupyter Notebook (27) and requires installation of the required packages on a local machine.

Users can upload a FASTA file containing their query sequence to obtain a detailed output in table format, where each residue is aligned to OXA-48 and annotated with standard numbering. Detailed step-by-step instructions and remarks are provided in Tutorial S4. The numbering system is also being implemented in the β-Lactamase Database (bldb.eu) for broader usability. Table ST5 lists all DBL sequences in the database (as downloaded in September 2024) aligned to the hmm profile.

## RESULTS AND DISCUSSION

### Secondary structure annotation

All class D β-lactamases share an α-β-α sandwich architecture with minimal deviation in the core structure. Structural variation arises primarily from insertions and deletions, particularly in the loops connecting the core elements. In the mature enzymes (i.e., sequences generated after post-translational removal of the signal peptide responsible for periplasmic export), some subfamilies feature an N-terminal β-strand. This additional strand forms backbone hydrogen bonds with the central β-sheet, effectively extending it within the protein structure. In other subfamilies, the N-terminus instead forms a short coil preceding the first α-helix, resulting in a central β-sheet composed of only six strands. Sometimes, structures of the same enzyme resolved under different experimental conditions vary, as in the case of OXA-23 (28–30). These variations also contribute to inconsistencies in the naming of secondary structural elements across different enzymes in the literature. Moreover, the naming of α-helices has been a recurring source of ambiguity, complicating cross-comparisons of structural data. For instance, work by Toth et al. resolved the crystal structure of OXA-143 (PDB: 5IY2) and identified 10 α-helices, 10 β-strands, and one 3_10_ helix excluded from sequential numbering. They referred to the Ω-loop as the loop between helix α6 and helix α8 (31). On the other hand, Antunes et al., analyzing the same PDB structure, reported 11 α-helices and six antiparallel β-strands (numbered starting from β2) and described the Ω-loop as located between helix G and helix I (the seventh and eighth helices) as they assigned ABC nomenclature to the helices (32). Such discrepancies highlight the need for a unified nomenclature for structurally homologous regions in class D β-lactamases (DBLs).

**Fig 2:**
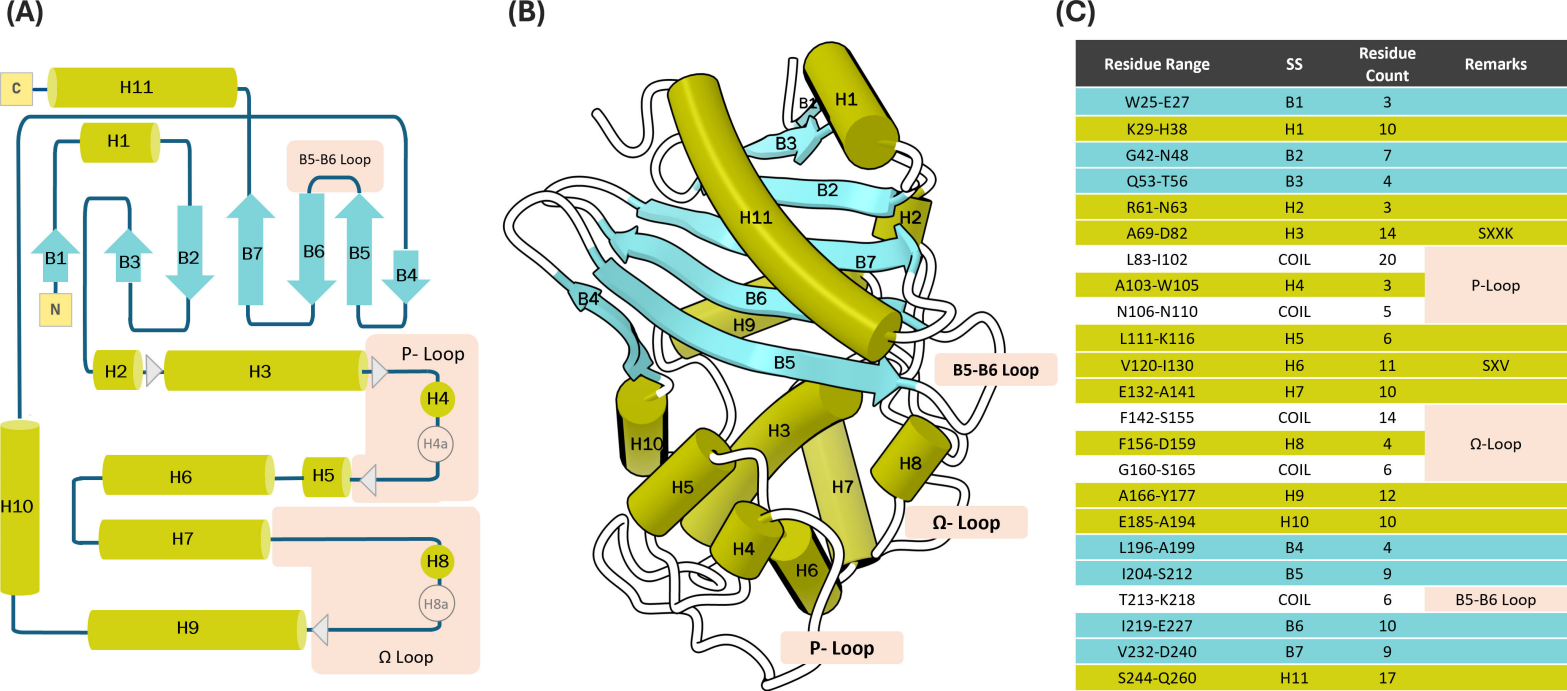
Secondary structure assignment for class D β-lactamases. (**A**) Flat diagram with consensus annotation of secondary structure elements of class D β-lactamases. Putative variable elements (shown in light gray) represent assignments that are transient or not conserved across the majority of the class D β-lactamase family. (**B**) Secondary structure annotation mapped onto the 3D structure of OXA-48 (PDB ID: 5DTK (22). (**C**) Table with residues bordering major secondary structure elements of OXA-48 as assigned according to the DSSP algorithm. An illustration of the secondary structural alignment of representative DBLs can be found in Fig. S2.

In our consensus annotation, β-strands and both α/3_10_ helices were numbered sequentially using the prefixes B and H, starting from 1. This alignment yielded a consensus of seven β-strands and 11 helices. Notably, the P-loop and Ω-loop, in many DBLs, include a 3_10_ helix, designated as H4 and H8, respectively. In cases where an additional 3_10_ helix is observed, a letter suffix is added to the helix name in the consensus annotation (e.g., H4a, H4b). Assignment of secondary structure using other algorithms (e.g., STRIDE) has resulted in the identification of some short β-strands in long loops. Such structures have been reported earlier to have 10 β-strands (31). To test the stability of these short strands, we further conducted temporal structural analyses using the STRIDE algorithm, which combines the use of hydrogen bond energy and statistically derived backbone torsional angle information when assigning secondary structure (33). Tracking these secondary structure elements over the course of 20 µs molecular dynamics simulation time reveals that these are transient structural elements and are not detected by conventional structural analysis tools (Fig. S3). To accommodate such elements in our annotation scheme, we advise annotating them with lowercase b for β-strands and lowercase h for helices followed by the numbers of the adjacent consensus elements. For instance, the strands near the edges of the P-loop should be called b34 and b45 as they lie between H3/H4 and H4/H5, respectively. When individual elements are absent in certain enzymes, they are omitted without altering the overall numbering order. Detailed illustrations of the consensus annotation and alignment are provided in Fig. 2 and Fig. S2.

### Conclusions

In the context of enzyme families with extensive sequence diversity, such as class D β-lactamases, a unified residue numbering scheme is essential for facilitating residue-specific comparisons across studies. A consistent approach that integrates sequence and secondary structure elements is particularly important in the era of advanced natural language processing (NLP) and biomedical literature mining. These technologies have become invaluable in accelerating drug discovery by enabling the efficient extraction and analysis of vast scientific data sets, especially in rapidly evolving fields like antimicrobial resistance (AMR) and pandemic research. (34, 35)

We emphasise that the scheme proposed here, termed structural alignment-based numbering of DBLs (SAND), is not intended to replace the unique residue numbering of individual proteins. Instead, it serves as a standardized reference point for molecular studies, enabling the straightforward comparison of homologous residues across enzymes and studies, particularly in figures and tables that highlight key residues or interactions. Alongside, we provide a consensus annotation to label the conserved secondary structure elements across this class of enzymes, addressing inconsistencies caused by variations in structure determination and assignment algorithms. While our automated workflow reliably identifies conserved motifs and aligns them to the reference in a simple output file, we acknowledge that the precise boundaries or identification of transient or additional secondary structure elements may differ. To ensure flexibility, our approach allows researchers to adopt the assignment method that best suits their needs while maintaining consistency with the broader literature. By simplifying cross-study comparisons and emphasizing conserved motifs, the SAND system aims to streamline research on DBLs and enhance accessibility by researchers from different disciplines.

## Data Availability

All data for supplementary information including the code, tutorial and sample files to run locally can be downloaded from https://github.com/shozebhaider/OXA-SAND.

## SUPPLEMENTAL MATERIAL

Supplemental figures aac.00150-25-s0001.pdf Fig. S1 to S3.

Supplemental material aac.00150-25-s0002.pdf A guide to use the automated workflow to assign SAND.

Table S1 aac.00150-25-s0003.xlsx A list of PDB structures used in this study.

Table S2 aac.00150-25-s0004.xlsx Structure-based sequence alignment with secondary structure annotations.

Table S3 aac.00150-25-s0005.xlsx DBLs sequences aligned to the built HMM profile.

ASM does not own the copyrights to Supplemental Material that may be linked to, or accessed through, an article. The authors have granted ASM a non-exclusive, world-wide license to publish the Supplemental Material files. Please contact the corresponding author directly for reuse.
