# Supplementary material for: SAND: a comprehensive annotation of class D β-lactamases using structural alignment-based numbering
Source: Antimicrob Agents Chemother. 2025 May 27;69(7):e00150-25. doi: 10.1128/aac.00150-25 (PMC12217458; doi:10.1128/aac.00150-25)
Supplement: Supplemental figures — Fig. S1 to S3. [file aac.00150-25-s0001.pdf]

SAND: A Comprehensive Annotation of Class D β-lactamases Using Structural Alignment-based Numbering

Fedaa Attana et al.

Supplementary Information

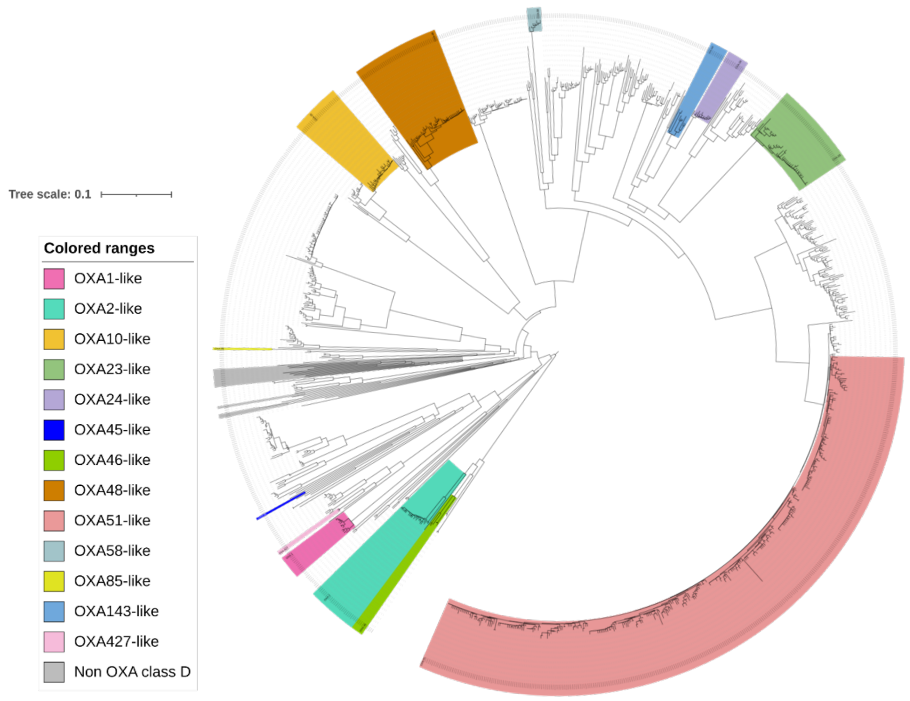

Fig. S1: Sequence alignment based phylogenetic tree of all class D enzymes documented in BLDB. Subfamilies with members for which 3D structures have been resolved experimentally are coloured according to the legend on the left.

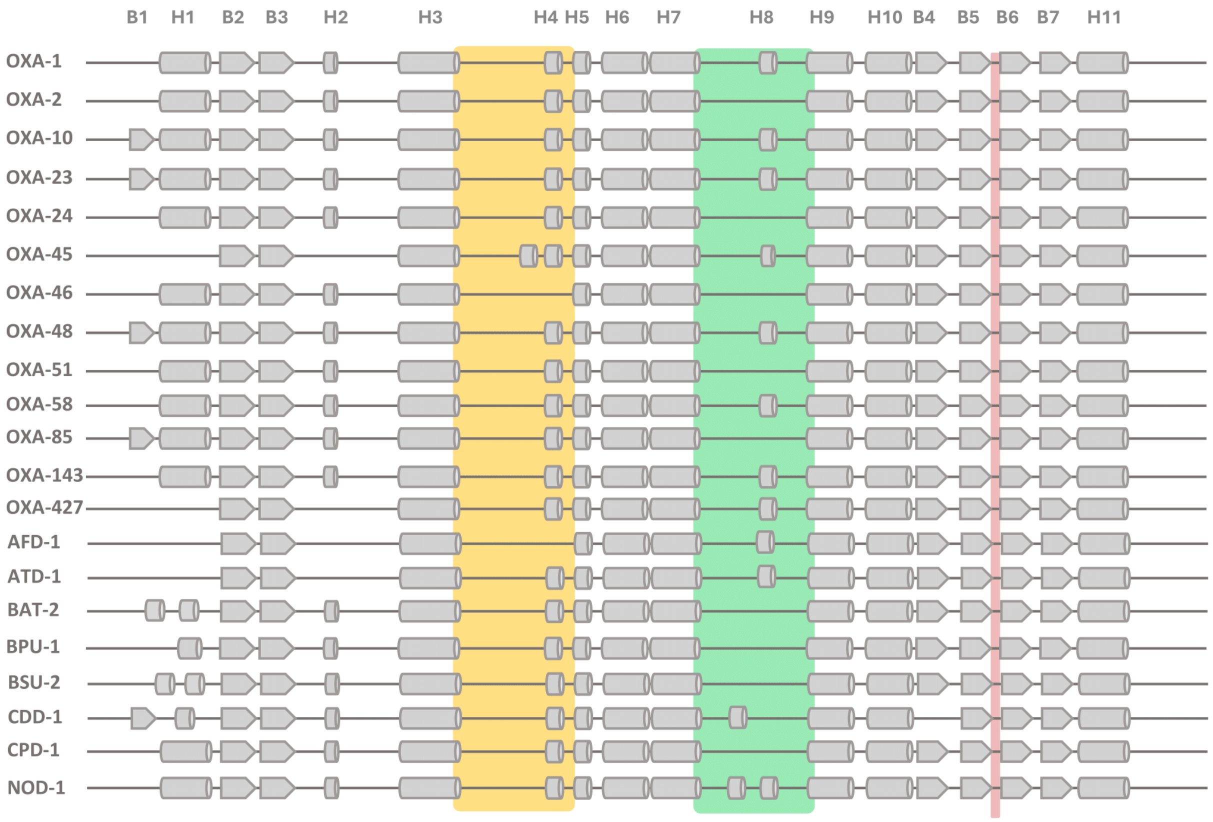

Fig. S2: Alignment of DBL secondary structure elements. Alignment based on tertiary structure homology among the 21 representative DBLs annotated according to the proposed consensus class-D secondary structure annotation. β strands are named as B#, Helices (α,3_{10}) are named as H#. Loops framing the active site: The P-loop, Ω-loop and B5B6 loop are highlighted in yellow, green, and red, respectively. In the case of BAT-2 and BSU-2, the first 2 helices align with consensus H1 helix hence are named H1a and H1b respectively. Similarly, NOD-1 has H8a in addition to the commonly putative H8 helix. Full alignment of the 21 structures is in Table ST3.

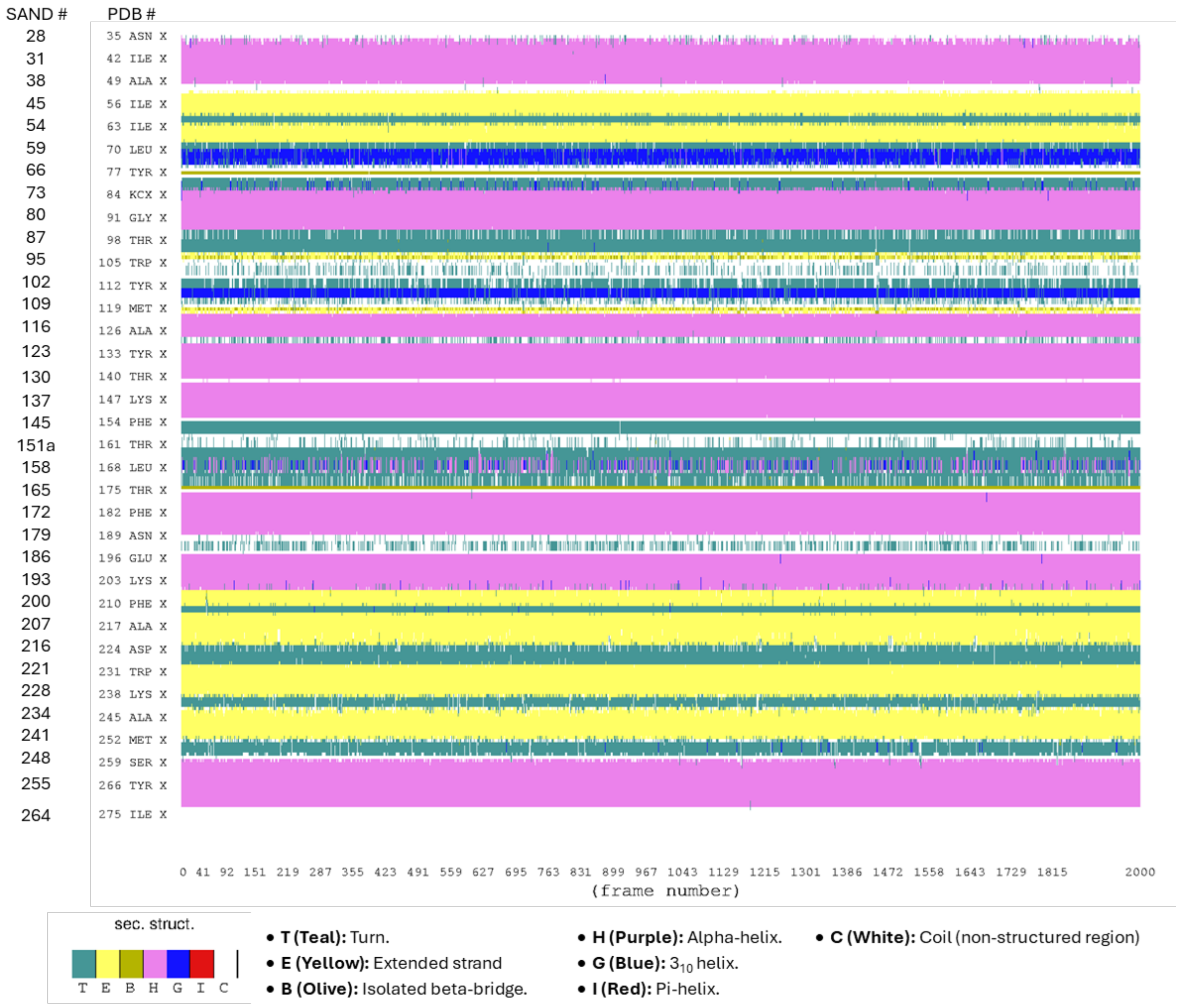

Fig. S3: Secondary structure evolution of the OXA-143 protein (PDB ID: 5IY2) over 20 μs molecular dynamics simulation. The data was sampled every 100 frames (2000 frames total) and analysed using the STRIDE algorithm via the VMD timeline plugin. Each row corresponds to a single residue, with secondary structure assignments indicated by the color scheme shown in the figure legend. The x-axis represents the frame number, while the y-axis lists residue indices as in SAND and OXA-143 PDB numbering.
