# Supplementary material for: SAND: a comprehensive annotation of class D β-lactamases using structural alignment-based numbering
Source: Antimicrob Agents Chemother. 2025 May 27;69(7):e00150-25. doi: 10.1128/aac.00150-25 (PMC12217458; doi:10.1128/aac.00150-25)
Supplement: Supplemental material — A guide to use the automated workflow to assign SAND. [file aac.00150-25-s0002.pdf]

Tutorial: A guide to use the automated workflow to assign SAND

This code handles aligning a query sequence to HMM profile pre-built with structure-based sequence alignment. Then mapping the standard numbering to the query. If you have no previous experience with python and installing packages on Linux we recommend you use the online version as it only requires logging into your google account.

Instructions to run ASSIGN_SAND online:

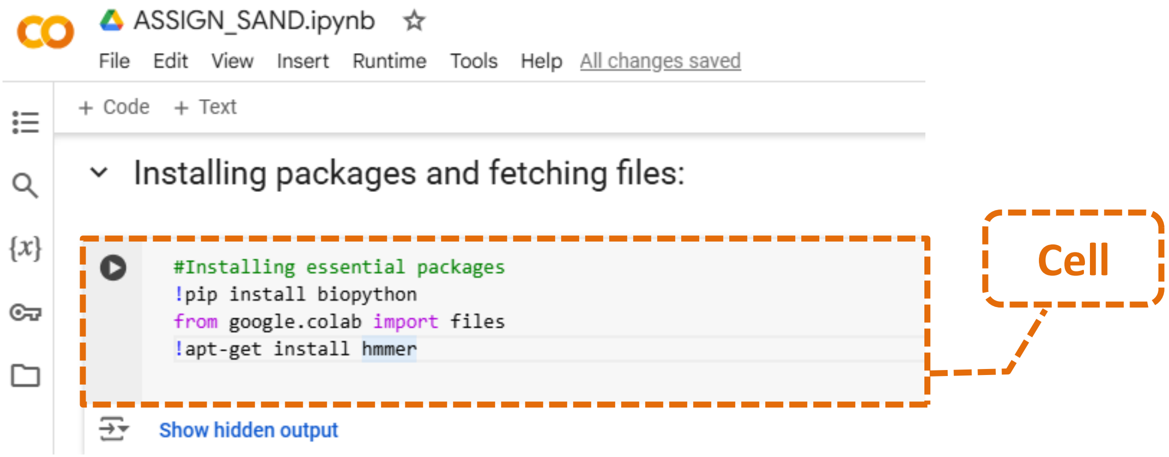

1. Open the Google-Colab notebook from the link:

   https://colab.research.google.com/drive/154cxz4MqYqLoo4gs-9i3fnk6uhv8tEsm?usp=sharing

2. Execute the cells (ie. click on the (►) symbol to the left of each cell) - The first two cells will take less than 2 minute to install necessary packages and files.

3. Once you run the main function you will be prompted to browse your device and upload your query .fasta file (In the attached folder we provided OXA-10.fasta as example)

4. Immediately, an interactive table will show up below the cell notebook and mapped_OXA-10_column.csv file will be saved to your Downloads folder automatically. OXA-10 in the file name is picked from the fasta file header uploaded.

5. If the download of the csv file does not initiate automatically, you might need to accept a pop-up message telling you that a website is trying to download files to your machine
6. You can open the file using any software that opens spreadsheets like MS Excel

Instructions to run ASSIGN_SAND on your local computer:

1. Download the zip folder named as S5 that has the required files and the Jupyter-notebook.
2. Install required packages as stated in the REQUIREMENT.txt file
3. Open the ASSIGN_SAND.ipynb file
4. Run the cells and type the path to your fasta file when you get prompt to do that. You will get your output immediately into your working directory.

Output:

The output will be of this outline:

Reference residue number	Reference Secondary Structure Annotation	Reference residue name	Query residue name	Query original numbering	Query Standard numbering	Comments
41		Q	N	38	41	
42	B2	G	G	39	42	
43	B2	V	V	40	43	
44	B2	V	F	41	44	
45	B2	V	V	42	45	
46	B2	L	L	43	46	
47	B2	W	C	44	47	
48	B2	N	K	45	48	
49		E	S	46	49	
50		N	S	47	50	
51		k	s	48	51	
52		Q	K	49	52	
53	B3	Q	S	50	53	
54	B3	G	C	51	54	
55	B3	F	A	52	55	
56	B3	T	T	53	56	
57		N	N	54	57	
58		N	D	55	58	
59	H2	L	L	56	59	
60	H2	K	A	57	60	
61	H2	R	R	58	61	
62	H2	A	A	59	62	
63	H2	N	S	60	63	
64		Q	K	61	64	
65		A	E	62	65	
66		F	Y	63	66	
67		L	L	64	67	
68		P	P	65	68	
69	H3	A	A	66	69	
70	H3	S	S	67	70	
71	H3	T	T	68	71	
72	H3	F	F	69	72	
73	H3	K	K	70	73	
74	H3	I	I	71	74	
75	H3	P	P	72	75	
76	H3	N	N	73	76	
77	H3	S	A	74	77	
78	H3	L	I	75	78	
79	H3	I	I	76	79	
80	H3	A	G	77	80	
81	H3	L	L	78	81	
82	H3	D	E	79	82	
83		L	T	80	83	
84		G	G	81	84	
85		V	V	82	85	
[illegible]		[illegible]	[illegible]	[illegible]	[illegible]	

Remarks:

- Look at the fifth and sixth column to know SAND numbering assigned to each of your query residue.
- Conserved residues are aligned with very high certainty
- Residues from Signal peptide region are not aligned as they are not part of the structure of the mature enzyme
- As mentioned in the main text, DBLs vary in loop sequences. Due to some loops' flexibility, structural alignment is not certain. Also, N and C Termini and first peripheral secondary structure element in some enzymes may not align with high precision Therefore, we recommend referring to the query structure or a structure from same subfamily to check if insertion-deletion sites are assigned in agreement to your study results
- For the same reason mentioned above, pairs of (insertion-deletions) might show up and need observing the structure. See in the example below: If the query S245 is closer to be part of the helix H11, then aligning it with D245 in the reference is better choice

243		T	N	243	243		
244		S	E	244	244		
-		-	S	245	244a	Insertion	
245	H11	D	-	-	-	Deletion	
246	H11	G	K	246	246		
247	H11	L	L	247	247		

Manual adjustment

243		T	N	243	243		
244		S	E	244	244		
-		-	-	-	-		
245	H11	D	S	245	245		
246	H11	G	K	246	246		
247	H11	L	L	247	247		
